# Abstracts and Gleanings

**Published:** 1883-06-20

**Authors:** 


### ABSTRACTS AND GLEANINGS.

**Hot Water in Post-Partum Hemorrhage.**—Dr. Parish, in a paper read before the Philadelphia County Medical Society (Med. Times) remarks :

I come now to a remedy not so long in vogue, but one that has in my hands given most satisfaction. I refer to hot water. Freely injected into the uterus at a temperature of about 112° or 115° F., it is a very prompt excitant of uterine retraction.

It will produce this effect with greater promptness than will cold water or ice, and will bring about uterine condensation after ice has failed because of the patient's great exhaustion. It should also be applied externally over the abdomen. I am in the habit of folding a towel as a flat compress, saturating it with water as hot as my hands will possibly endure, and, squeezing out the redundant water, to apply the wet and hot towel on the abdomen, and then, placing my hand on the towel through it, to compress the uterus. The womb will be felt to harden promptly even when the same manœuvre with iced water has previously not succeeded in obtaining condensation. The remedy may be rendered disinfectant with permanganate of potash, carbolic acid, etc., or even more efficient in producing retraction by the addition of vinegar, though I have never seen this addition necessitated. Hot water controls bleeding from the placental site by securing with certainty prompt and complete condensation. It has no, or at least very little, influence in the formation of thrombi ; it washes out all clots and leaves the uterus clean ; it will secure condensation after other agents have, by reason of the patient's exhaustion, failed ; it is operative when the patient is under ordinary etherization ; it controls bleeding from lacerations at the same time that it controls the loss of blood from the placental area ; it controls bleeding from the laceration by producing contraction of the vessels, and not by causing coagulation of the blood ; it is more invariably attainable than other agents, and can be had in all seasons and in all climates.

The water should be hot enough—as hot as the hand, or, better, the uncovered arm, can endure. This degree of heat is not usually painful to the patient, excepting to a limited extent at the external genitals. There is no shock resulting from its use ; it is a remedy against shock. It is followed by an increased feeling of comfort. It will regulate irregular uterine contraction by stimulating the entire body into contraction, and will thus check bleeding when superinduced by paralysis of the placental area. It is valuable in the prevention of hemorrhage in "bleeders," or in any case in which there is reason to expect undue loss of blood.

Of all the remedies directed towards the stopping of post-partum hemorrhage, hot water thus combines the maximum of promptness and certainty with the minimum of noxiousness to the patient.

To summarize very briefly the line of management, I would say, as soon as the child is born, secure, with the hand over the abdomen, retraction about the placenta; if hemorrhage occurs, express the placenta and continue with the hand externally to effect retraction. If the bleeding does not promptly stop, inject hot water in considerable quantities into the cavity of the uterus. While the injection is being effected, apply or have applied at the same time a hot wet compress over the abdomen, and, by a hand placed on this compress, make firm pressure on the uterus. Ergot should be, as soon as possible, injected, hypodermically, but the above measure should take precedence in time of application:

[℞ Ergotin.................................15 grains,

Water.................................6½ drachms,

Glycerin.................................6½ drachms,

Dose, one drachm hypodermically.—ED. REC.]

In post-partum hemorrhage dependent upon a laceration of a cancerous cervix, I have had of late, no opportunity to use the hot water. In such a laceration it should be employed; but if the bleeding is not quickly controlled, a piece of cloth saturated with Monsel's solution (sol. persulphate of iron) should be pressed with the fingers against the bleeding surfaces. In post-partum bleeding following placenta prævia I have not used hot water, but should expect its favorable action. In one such case, swabbing the placental area around the internal os with Monsel's solution acted so promptly in preventing death that I should prefer at once to resort to this remedy in such cases. I should never make an application of Monsel's solution by way of merely preventing hemorrhage even in cancer of the neck, nor in placenta prævia; for bleeding after labor is not an invariable accompaniment of either of these conditions.

There are not a few minor adjuvants in the treatment of post-partum hemorrhage, such as the compression of the aorta, lowering the head, admitting fresh air, etc., which are more or less valuable, but which do not demand discussion in this paper.

In cerebral anæmia following an excessive loss of blood, the hypodermic injection of stimulants—whisky, ether, etc.—is of very special value, as absorption in syncope from the stomach is very slow or in total abeyance.

Small doses of an opiate in filling the cerebral vessels, diminish shock, and quiet restlessness.

DISCUSSION ON DR. PARISH'S PAPER.

Dr. William T. Taylor said that he had, during the earlier years of his practice, been afraid to introduce the hand into the uterus; it was considered dangerous; for it was a kind of *noli me tangere*, whose cavity must not be invaded. He had, however, changed his mind, and now regarded the hand as really the first thing which was commonly "on hand" for use in post-partum hemorrhage and other troubles. For, if the child had recently passed through the os uteri, it was not generally so much contracted but that the hand could be conveniently and carefully introduced in order to remove the cause of the hemorrhage, clots, etc., and then, by pressure externally, the uterus will contract and expel the hand, when the hemorrhage will cease.

Dr. Ludlow inquired whether Monsel's solution produced a true coagulation. He thought it was rather a carbonization. He would like Dr. Smith to state whether he knew of any death resulting from the use of the iron hæmostatics, particularly Monsel's solution, the only one he had used. He had been one of the first to use Monsel's solution in these cases, and had used it often, by means of a special instrument of his own make, there being no other at that time to be had, and never had any trouble. Dr. Parish himself, in the paper, had spoken of it as a last resort. Why last resort ? He recalled a case where a patient had been bleeding for some time, and in which ergot, ice, and other methods had failed. He had never used Monsel's salts in so recent a case before (this, it must be noted, was before hot water was suggested), but he used it here with success. He would not use it always, of course, but was not afraid of the kind of coagulation produced, which he believed to be a direct chemical change by the hæmostatic upon the blood. Our chemists can soon determine this. Besides, by the most of means used coagulation is produced.

Dr. Ludlow said that he was aware that deaths had occurred from the use of Monsel's salt, but he had referred in his question only to its use, as Dr. Barnes had advised, after the uterus had been emptied. It was only under such circumstances he used it. He very soon found that it would not permanently stop the flowing of blood when even the small papilloma, not larger than a usual wart, existed in the uterus. The abuse of an article, or a wrong mode of using it, or the unskillful performance of an operation, is no argument at all against its proper use. He advised Dr. Smith to try the iron hæmostatics and report the result. Dr. Ludlow said that he could detail numerous cases of hemorrhage controlled by iron hæmostatics when every other means had failed. It is dirty, undoubtedly ; but that can be no objection when life is involved.

Dr. Wm. S. Stewart was an advocate of the use of iron. He had not seen any bad results from it. He never allowed the clots to remain, but as soon as the patient had reacted he removed the clots and syringed the uterine cavity, repeating this latter operation for several days. He was glad to learn that this system of syringing had been adopted at the Woman's Hospital. The use of iron and hot water will be rarely necessary if labor is properly conducted and not hastened ; but no clots or shreds must be allowed to remain. If we carelessly pass over these, we will have profuse hemorrhage, which will require strong treatment. We should explore the uterus thoroughly, examining to the fundus. If the nails are trimmed and the hands clean, no injury will be done. He had sometimes been obliged actually to scrape off the placenta, but had seen no harm result.

Dr. Hewson had recently seen Dr. J. Marion Sims perform the operation of incision of the os uteri, and, suspecting that the circular vessels might be wounded, Dr. Sims had used cotton saturated with dilute tincture of chloride of iron, one part to four, and packed it well into the vagina, back of the cervix. The application was considered on the occasion by Dr. Sims the best antiseptic and styptic.

Dr. Blackwood agreed with Dr. Parish's advice to retain the placenta fifteen or twenty minutes. He thought that the vessels of the placental site are filled with clots. He had made a post-mortem examination of the body of a woman who had died of pulmonary hemorrhage just after the third stage of labor, and he had found the uterine vessels filled with clots. He did not think that the hand, if carefully introduced into the uterine cavity, could do harm. He often did it, placing the other hand at the same time on the abdomen. In treating hemorrhage, he had used vinegar and lemon-juice, but did not like ice. Hot water is the remedy, but in one case he had seen decided shock from its use. He did not believe in iron.

Dr. Welch thought that Dr. Parish had overestimated the danger of introducing the hand into the uterus, which is always greatly relaxed and offers but little resistance to such introduction. It is necessary to insert the hand in order to discover the cause of the hemorrhage. If it results from laceration, that condition can be recognized, and if the uterus contain clots we can turn them out. The late Dr. Charles D. Meigs used to insist upon this both in his lectures and his writings.

Dr. Welch wished to emphasize the advantage in the use of vinegar. He had seen a case of post-partum hemorrhage in which the bleeding had been very extensive; the color of the lips had even been lost, and the patient could only speak in a whisper. The hand was introduced and the clots turned out, but this did no good. Ice was also uzed, without effect. The patient was so far gone that she did not even feel the ice within the uterus. A clean white rag was saturated with vinegar and introduced, and in an instant the uterus contracted. The patient rallied, and no further hemorrhage occurred.—*Med. Times.*

**Clinical Notes on Opium Addiction.**—Dr. J. B. Mattison, (Soc. Co. of Kings) reports:

G. H., physician, æt. 42; ten years addiction; daily taking, 18 grs. morphia, hypodermically; cause, peritonitis; effects, bowel torpor—for years no evacuation without enemas; vesical and sexual debility, anorexia, indigestion, hemorrhoids—the hemorrhage sometimes profuse, mental depression, muscular weakness and emaciation; in general, a wreck-like state of mind and body. On admission was pallid and weak. Tonic regime, exclusively—strychnine, iron and digitalis, with generous diet for ten days. Morphia then reduced to six grains without discomfort. Sedative treatment secured desired effect, and entire opiate withdrawal in eight days. During afternoon of last day's habituation—the final dose being one-third of a grain—had severe headache, of limited duration, relieved by hot sitz bath and cold to the head. Moderate restlessness followed, subsiding in 48 hours. No other symptom of note. No vomiting; no diarrhœa. Patient made an astonishingly rapid recovery, and was dismissed, cured, on the thirty-first day of his special treatment.

Later, per letter, he says: "Do you ask, 'does the old enemy ever assert power, and tempt me to the hypodermic?' Most gladly can I answer, no! There is not the least physical desire for morphia!" Still further testimony to his radical recovery is his present active pursuit of his calling.

The therapeutics of these cases included bromide of sodium, hot baths, electricity—both galvanic and faradic currents, atropia, strychnia, hyoscyamia, quinia, chloral, coca, cannabis indica, Jamaica dogwood, varied tonics, full feeding, and cheerful surroundings.

To note these in detail requires some preliminary reference to the morbid condition they are intended to relieve. The symptomatology of opium abandonment, in our opinion, relates to an exalted activity of the spinal cord manifested in varied reflex irritations. To this are attributable the aches, pains, vomiting, purging, collapse and horrible discomfort, in general, which follow entire and abrupt withdrawal of a long accustomed opiate. If this be correct, it is also correct to assert that any drug able to control this over-action must prove potent for good in treatment. Such we have in the bromides. Their power to subdue reflex irritation is known to all, and in no disorder is this more happily proven than in the one to which we refer.

A special and original application of this power is what we term *preliminary sedation*, which consists in the giving of the bromide for a time *prior* to entire opiate withdrawal—meanwhile gradually reducing the accustomed narcotic—so that at the time of maximum spinal irritation we have maximum bromide sedation, and the one counteracts and controls the other.

We use, exclusively, bromide of sodium. It has two leading advantages. Saving bromide of lithium, it contains the largest proportion of bromine, which is the active factor, and it is less unpleasant than any other, never, in our experience, causing gastric trouble. Minor points in its favor are, lessened tendency to digestive and muscular impairment, and cutaneous irritation.

We use it in full doses—60 grains, increased to 100 or 120—in eight ounces of water, twice daily, at twelve hour intervals, and continue it from five to ten days, or even longer—average time, one week—the extent of its giving, both amount and duration, depending entirely on the peculiarities of each case, before and during treatment.

Hot baths, 110° to 112°, are the most efficient agent at command to relieve and remove the peculiar restlessness which is an *invariable* sequel of opiate abandonment. They are given often as required, ten to twenty minutes duration. Their efficacy is sometimes enhanced by a short douche or shower.

Electricity is used as a tonic and sedative. The galvanic current we often employ from the outset, and, after abandonment, find it useful as a general restorative and remover of local pains. For the muscular debility following withdrawal, nothing, in our experience, equals general faradization—10 to 20 minute seances daily. The sense of exhilirating comfort resulting is often very decided. Occasionally it is used twice daily, and, very exceptionally, it is not at all acceptable.

Atropia is used in initial doses of 1-120 gr., hypodermically ter die—or its equivalent by the mouth—and pushed until it produces systematic effects—dry throat and disturbed vision.—This has never required a dose exceeding 1-40 of a grain.

Strychnia is given in subcutaneous doses of 1-30 of a gr., thrice daily, and continued, in some form, throughout treatment.

Hyoscyamia, in our experience, has proven itself the nearest approach to morphia of any alkaloid yet presented. We use Merck's *amorphous*, in the dose of one-sixth of a grain hypodermically, and have known it, repeatedly, to produce steady sleep of several hours' duration.

Quinia is used for a two-fold purpose—tonic and sedative. As the former, in two grain doses, three or four times daily, throughout treatment. As a sedative, in 20 grain doses, given a few hours in advance of the restlessness following withdrawal, and repeated at 12 to 24 hour intervals, as required. Thermometric observation proves its power to control the rise in temperature noted after opiate abandonment. Subsequently, it is sometimes given as a soporific, and its efficacy, in this respect is, to us, beyond dispute.

During the first three or four days after opiate discontinuance, chloral fails of its usual effect, and we never employ it. We have not noted the excitement, stated by Levenstein, but, simply, that it does not induce sleep. Subsequently, as a hypnotic, it answers every purpose, and is given—usually combined with a bromide or hyoscyamus—as long as may be required. We use Squibb's make, in decided doses, our experience being that a single full dose is preferable to one small and frequently repeated. When acceptable to the stomach it is often kindly received, per rectum, same dose as by the mouth, in an ounce or half ounce of warm mucilage.

Cocoa, though far from being what some theoretical enthusiasts have claimed, is a stimulant of value, and as such fills a place in treatment. We use Squibb's extract, in half-ounce doses, frequently repeated after the opiate withdrawal.

Cannabis indica, in some respects, is an efficient substitute for opium. It relieves pain and brings sleep, though often causing a mild, harmless intoxication. After a trial of various preparations, foreign and domestic, we prefer the fluid extract made by Squibb. It must be given in large doses, the ordinary dose of the books being of no avail whatever.

Jamaica dogwood is a somewhat uncertain anodyne and soporific, yet worthy of trial to relieve the neuralgic sequelæ of opium addiction. We give it in full ounce doses.

Varied tonics include iron, arsenic, digitalis and cod-liver oil. The first two if anemic. Digitalis after the sedative treatment, as a tonic and also diuretic, to eliminate the bromine. Cod-liver oil is a particularly valuable roborant, possessed of special nutrient properties to repair the wear and tear of prolonged narcotic addiction. We prefer Moller's plain oil and Phillips' emulsion.

During the first two days of opium abstinence, patients are best restricted to a diet of milk and lime-water, in small amounts, often repeated. After that, full feeding is allowed and encouraged to the largest extent consistent with gastric comfort.

**Ulceration of the Os Uteri.**—A writer in American Medical Journal, June, 1883, says:

"When we suspect that a woman is suffering from ulceration of the uterus, we propose an examination and forthwith make it, if permitted. We use one of Staufer's speculums, with hollow bulb conductor, stem and check wheel. These speculums are easily introduced, and when in place the vaginal portion of the uterus is well exposed and readily examined.

If we find an ulcerated os, we make the required local application through this same speculum, as follows: ℞ S. H. Kennedy's concentrated aqueous extract of pinus canadensis (dark), one teaspoonful; warm water, one tablespoonful. M. Saturate a wad of cotton batting with this solution, and while the speculum is in place, introduce the saturated cotton through it. That the medicated cotton may be placed firmly upon the ulcerated and inflamed os, we put the hollow bulb conductor into the speculum, and with this we push the cotton entirely through the speculum, and against the uterus. We now carefully withdraw the instrument, leaving the medicated cotton in place. This application may be repeated daily, and after a few times the patient can introduce the speculum and apply the medicine without assistance. As improvement takes place the solution can be made weaker.

For making such examination and application, no speculums equal these. And for local treatment, in such cases, nothing equals S. H. Kennedy's concentrated aqueous extract of pinus canadensis. Richardson & Co., of this city (St. Louis) furnish it in either form required, dark or white.

It is astonishing how rapidly vaginal and uterine inflammation subside under this plan of treatment. Tender parts grow less sensitive, itchings and smartings are relieved, prolapsus disappears in many cases, leucorrheas are cured, and a general change for the better is enjoyed.

**Bovine Virus Disapproved.**—Dr. Varian, in Medical and Surgical Reporter, in an address before the Medical Society of Pennsylvania, says:

The uncertainty of the action of animal virus, and the frequently recurring necessity for its renewal, I found wearisome and discouraging. The brief period which elapsed after a successful vaccination before the subject became susceptible to a re-vaccination argued a weak prophylactic power, which argument was not weakened by the occasional occurrence of varioloid shortly after a successful vaccination. But I still persevered, for I felt it my duty to give my patients what was considered the purest and best means of prophylaxis that could be obtained. Still I could not but feel that the entire failure of at least thirty per cent. of all my primary vaccinations was discouraging, and the necessity of going over the same ground again and again was, to speak mildly, monotonous.

During the winter of 1881 and 1882, small-pox was epidemic, and its loathsome wave rolled over the entire country. Almost every individual in my section demanded vaccination, and it was necessary to obtain material from every source of supply within my reach. The result in many cases was the production of an unhealthy and poisonous sore, often phagadænic, and always inflammatory, which gave great trouble and frequently took weeks to heal. These cases all suffered from severe systemic disturbance, which in some cases was not without danger to life. When finally healed, the cicatrix in some cases became the seat of an erectile tumor which resisted all measures of destruction, and was finally removed by extirpation with the knife. Had this experience been personal to myself alone, it would have proved disastrous to my practice and reputation; but it was a common experience to all practitioners in my neighborhood; and the universality of the misfortune was our protection from public reprobation. An experience of nearly thirty years use of humanized virus had shown me that it possessed great protective power against variola, together with certainty of action, and freedom from such effects as above described. Less than ten years' use of animal virus has led me to believe that it is uncertain in its action, that its protective power is not so lasting, and that at times it was not free from a liability to produce disastrous effects. I have strong doubts whether any protection from variola has been obtained in those cases wherein the inflammatory sore above described was produced; but I have no doubt whatever, that it would now be difficult, if not impossible, to induce the sufferers to submit themselves to another vaccination. So far as I have collected the testimony of the medical men in my vicinity, their experience has been similar to my own, and their faith in animal virus has received a rude shock.

**New Remedy for Syphilis.**—The Medical Times and Gazette, January 6, 1883, says that Prof. Liebreich brought forward, at the last meeting but one of the Berlin Medical Society, a new drug for the treatment of syphilis by the subcutaneous method. This drug rejoices in the name of hydrargyrum formidatum, and, is, therefore, merely a different form of the old cure for syphilis. The mode of its preparation was not stated; chemically, it belongs to the amide group, in whose structure the monovalent amidogen ($NH_2$) plays an important part. Liebreich was led to think of this new preparation from the notion that the ordinary amides of the body, of which urea may be regarded as the principal one, pass out of the organism in an undecomposed state; when, however, an amide is in combination with a metal, decomposition readily occurs, and the metal is reduced and deposited. Liebreich repeated his experiments before the Society, and showed that these conjectures were quite true for the metal mercury. It is supposed, therefore, that the formamide of mercury, after the hypodermic injection, undergoes disintegration; and so the mercury is set free, and is able to exert its well-known power over the lesions of syphilis.

The preparation is easily soluble in water, is of neutral reaction, does not coagulate albumen, is not precipitated by caustic soda, and the presence of mercury can be demonstrated by means of sulphide of potassium. The drug, when injected under the skin, produces its effects very surely and rapidly. This is not regarded as a disadvantage, for the medicine is said to be easily borne, and has never produced salivation in Liebreich's hands. There is very little pain attendant on the injection, which has never excited any inflammation. From a half to a whole of a Pravaz syringeful (a one per cent. watery solution) may be injected twice or thrice daily. Liebreich looks on the preparation as the best we yet have for subcutaneous injection.—*Med. and Surg. Rep.*

**A Five-Franc Piece Swallowed.**—Dr. Pixley, in Medical and Surgical Reporter, says:

Miss Ellen S., aged 17, single, came to me for treatment in September, 1874, when the following facts were elicited: During a scuffle with a friend over the possession of a five-franc piece, she placed it in her mouth for safe-keeping, and inadvertantly swallowed it. The coin lodged at the cardiac orifice of the stomach, giving her much pain, nausea and vomiting. The patient came to my office about three hours after the accident, and I saw at once that I could not extract the coin; I therefore took a probang and pushed it into the stomach, employing considerable force in the operation. Vomiting of blood together with the contents of the stomach, at once followed, and continued at intervals for eighteen days, attended by emaciation and loss of strength and appetite.

Often during this time, whenever the coin changed its poisition, she would fall insensible, as though struck in the stomach by some instrument. The young lady frequently remarked that she "could nearly vomit up the money," and expressed the thought that it could be more easily done if the stomach were filled to repletion. She accordingly filled her stomach to its fullest capacity with pancakes, and made ready to try the experiment. The first attempt was successful, the coin being ejected with such force as to knock out the two upper incisor teeth, and to loosen one bi-cuspid so much that it soon fell out. Large quantities of blood, mixed with the contents of the stomach, were thrown up, and the digestive power was greatly impaired. This condition of affairs lasted twelve months; and during the first three months it was with great difficulty that food of any kind could be retained in the stomach. Up to this time the action of the stomach showed ulceration, by the vomiting of blood and pus. These attacks of vomiting became less frequent and severe, and at the end of six months the patient seemed in a fair way to recover, and is now (1883) well.

**Corrosive Sublimate.**—The Medical Press & Circular, January 31, 1883, says that this powerful drug, whose virtues as an antiseptic were first made known to the world by R. Koch, is now coming rapidly into use. Tarnier employs it freely in his maternity hospital. Every attendant on entering the labor wards must wash the hands and arms in a solution of corrosive sublimate (1 in 1,000). The patient's genitals are bathed in a solution of the strength of 1 in 2,000; this is also the strength required for vaginal injections. He appears to be well pleased with the results.

Billroth has also been employing it as a surgical dressing in a case of suspected anthrax. A patient admitted into the hospital had been in attendance on a sick ox, from the rectum of which he had removed masses of coagulated blood, passing the hand deeply into the cavity. Afterwards pustules made their appearance on the dorsum of the hand. It was this condition of the hand, with the attendant history, that led Billroth to a trial of corrosive sublimate, apparently of the strength of 1 in 5,000 No fever had set in after several days' employment of the antiseptic.—*Med. and Surg. Rep.*

**Treatment of Delirium Tremens.**—Death, no doubt, in delirium tremens arises from the want of sleep, but the want of sleep arises from want of nourishment. So says Dr. F. P. Atkinson, in The Practitioner, January, 1883. He recommends half a tin of Brand's liquid essence of beef and half a pint of milk to be taken alternately every two hours, and all stimulants to be cut off. Twenty-five grains of chloral, with thirty minims of compound tincture of cardamon in an ounce of water, every four hours, after the beef tea, will be useful. By this treatment, the patient is generally free from delusions in thirty-six hours; but good strong liquid food should be taken less frequently for several days. When there have been from ten to twelve hours more or less continuous sleep, then it is advisable to give up the chloral, and give thirty minims of the compound tincture of gentian with five minims of the tincture of nux vomica three times a day for about three days. This restores the tone of the nervous system and stomach, and creates an appetite. A little tincture of euonymin may next be substituted for the nux vomica, and some Carlsbad salt may be given in the morning when required.—*Med. and Surg. Rep.*

**Confidential Communications.**—The Northwestern Law Reporter reports the following decision of interest to physicians, since it involves the question whether information obtained by a physician from a patient, orally or from observation, must be disclosed on the witness-stand. The decision was given by the Supreme Court of Missouri. The law declares that "he shall be incompetent to testify concerning any information acquired by him from any patient whom he may be attending in a professional character, and which information was necessary to enable him to prescribe as a physician or operate as a surgeon." It was claimed that this statute referred only to what had been obtained from the patients verbally. The court held that, from the nature of the case, the result of the physician's observations were comprised under the term information, and that any other view would be destructive of the confidential relations of physician and patient. —*Gaillard's Med. Jour.*

**Removal of Warts.**—Dr. Unna, of Hamburg, (Monatschrift Prakt. Dermat., 3, 1882), had a young girl under his charge, whose dorsal surfaces of both hands were the seat of over a hundred warts, daily more making their appearance. He made on gauze tissue a plaster mass, which contained 10.0 grm. arsenic and 5.0 grm. of mercury. This was kept on during the night. Two weeks later all the warts had disappeared and the healthy skin was seen. The cure here is not established by necrosis and dropping off of the excrescences, but like nature's spontaneous cure, by absorption.

We ourselves used to make cauterization our main treatment for the removal of condylomata. A year ago, a patient came to us, who, besides suffering from icterus, due to catarrhal inflammation of the bile ducts, was affected with condylomata also. As the latter were very large, preventing the prepuce from being drawn forward, their removal was desirable. They gave rise to an annoying itching and to a fetid odor. Not wishing to operate on them while the patient still had the jaundice, we told him to dust the parts daily with—

℞ Hydrarg. muriat. mit............................ ʒj,
Acid borac. pulver.................................gr. x.
M. Fiat pulvis.

And were not a little astonished to find, three weeks later, not a sign of them left. They were all absorbed. Since then we have often had occasion to use this powder, and invariably with the same good success.—*Med. and Surg. Reporter.*

**Eucalyptus Globulus.**—Dr. Smith (Medical Tribune) in reporting an obstinate case of intermittent fever, says:

I had read of the virtues of the eucalyptus globulus, in just such cases, and I resolved to test the medicine upon this patient. In accordance with this resolution I wrote the following prescription:

℞ Fluid ext. eucalyptus globulus................ }
Glycerine......................................... } aa ℥iv.
Simple syrup.................................... }
M. Sig. One teaspoonful once in four hours.

She had no fever or perspiration after the first dose. I ordered her to continue the medicine until all was used. She was soon restored to her usual health. This prescription permanently cured her.

I soon had another opportunity of clinically testing the curative power of this medicine.

I was called in council to see a woman suffering with typho-malarial fever. The patient was 75 years of age, and had been under treatment for four weeks. At the time I first saw her she was greatly emaciated. The pulse was 110; temperature, 104; no appetite, headache, with extreme prostration. The fever was of the remittent type. I learned from her physician that all the usual anti-malarial, anti-periodic and fever remedies had been given her without any marked benefit. The disease was complicated with a congested condition of the lungs, cough, shortness of breath, etc. I advised an expectorant for the cough, when troublesome, and ten drops of the fluid extract of eucalyptus globulus to be given once in four hours, with the recommendation that it be increased one drop daily until fifteen drops had been reached, and then to continue it at that.

I met her physician daily, and watched the patient closely. The fever gradually subsided, and at the end of one week it had entirely disappeared. The patient rapidly convalesced. The attending physician and myself agreed that the cure was chiefly to be attributed to the eucalyptus given. I have treated many cases of different forms of fever with this remedy with like results. With these remarks upon the comparatively new remedy in fever, and thanking you for your patience in listening to the contents of this paper, I will close.

**A Foreign Body in the Air-Passages.**—Dr. A. E. Benthall, in British Medical Journal, relates a case which is of interest from the length of time the body was retained.

The patient, a strong, healthy man, had been shooting with a blow-pipe and dart, and when he was about to blow the dart from the tube, coughed suddenly, and by the force of the inspiration the dart was drawn into the mouth. The patient thought he had swallowed it, but Dr. Benthall, from the history of the case, etc., concluded it was lodged in the right bronchus. The dart was made of a strong needle two inches in length, around the blunt end of which a quantity of worsted was wrapped, sufficient to fit the half-inch calibre of the blow-pipe tube. For two days slight pain in the region of the bronchus was experienced, and a little cough with expectoration streaked with blood.

The third day he had a violent fit of coughing with slight hæmoptysis. He remained very quiet for a fortnight, but upon resuming work had violent fits of coughing every three or four days with slight hæmoptysis and a taste of worsted in his mouth. This continued at intervals for eight months, when a violent fit of coughing brought up the thick end of the needle with some of the worsted still attached to the eye, together with a little blood. The piece of needle about an inch in length he extracted himself from the roof of his mouth. Six hours after a similar fit of coughing brought up the point of the needle. The needle had rusted completely through the middle.—*Chicago Med. Review.*

[In a case which fell into our hands last summer of a watermelon seed in the larynx of a child, a wheezing and difficult respiration continued for a period of five weeks, when in a violent fit of coughing it was ejected ; and this will probably happen in a majority of cases wherein the foreign body is not too large to be tolerated. If the patient can breathe reasonably well the surgeon may often trust the case to nature, being ready, however, for prompt action in case of emergency.—ED. REC.]

**Diagnosis of Consumption.**—The following is the closing paragraph of a paper by Drs. Gradle and Woltmann on "The Diagnosis of Consumption by Means of the Microscope," read not long ago before the Chicago Medical Society, and giving a general review of the facts hitherto known, with the results of their own investigations—

The invariable conclusions from our own work, as well as that of other observers, are that every case of pulmonary tuberculosis can be diagnosed by means of microscopic examination of the sputum, even before the clinical examination reveals it with certainty; and that when repeated proper examination of the sputum fails to show the bacillus tuberculosis, pulmonary tuberculosis does not exist. To speak with certainty in any case requires, of course, that the observer should have familiarized himself with the methods and possess the proper appliances. Our success has been so invariable, that we feel confident enough to challenge the society to produce a case of tuberculosis in which we cannot demonstrate the bacilli.—*Pop. Sci. News.*

**Fistula Ani.**—Demure, in Wien. Med. Woch., records the case of a child, eight days old, with a very long and deep fissure of the anus. It caused great pain, and bled freely each time the bowels were moved. The irritation from the fissure produced chorea. The sore was dried carefully and painted freely with a mixture of 1 part iodoform, 4 parts balsam of tolu, and 20 parts ether. The ether evaporates and leaves the tolu as an insoluble varnish containing the iodoform. Complete recovery took place in eleven days.—*Can. Jour. Med. Sc., Dec.*

**Volta Prize.**—This prize of $10.000, instituted by a decree of June 11, 1882, will be awarded in 1887 for a discovery which will render electricity effective and economical, as a source of heat or light, or of chemical action, or mechanical power, or a means of transmitting dispatches, or treating invalids. Any one may compete up to June 30, 1887. The examining commission will be appointed by the French Minister of Public Instruction.—*Maryland Med. Jour.*

**Quinine Amaurosis.**—The characteristic features are: 1. Total blindness after taking a large quantity of quinine; 2. Pallor of the optic disks; 3. Marked diminution of the retinal blood-vessels, in number as well as in size; 4. Contraction of the field of vision. The *total* blindness is only temporary. Relapses appear to oceur, and from comparatively insignificant doses. Horizontal position seems to be beneficial.—*Knapp, Archives of Ophthalmology; Maryland Med. Journal.*

**Colic—Quinine.**—Dr. Derby says, in the Record, that ten grains of quinine, given in the early stages, will prevent an attack of intestinal colic, or, administered at any stage, will speedily effect a cure.—*Can. Pract., Jan.*

